# Exposure Conditions and Pharmacokinetic Principles: Interpreting Bisphenol A Absorption in the Canine Oral Cavity

**DOI:** 10.1289/ehp.1307424

**Published:** 2013-12-01

**Authors:** Justin G. Teeguarden, Jeffrey W. Fisher, Daniel R. Doerge

**Affiliations:** 1 Pacific Northwest National Laboratory, Richland, Washington, USA; 2 National Center for Toxicological Research, U.S. Food and Drug Administration, Jefferson, Arkansas, USA, E-mail: jt@pnl.gov

Gayrard et al. (2013) reported significant (~ 80%) absorption of bisphenol A (BPA) from solutions applied to the oral cavity of dogs, leading to higher serum BPA (aglycone) concentrations than occurred when BPA was absorbed through the gastrointestinal tract. This finding is consistent with first principles and experience with orally absorbed drugs. The implications for human exposure and health will be clear when experimental evidence is available regarding the extent and frequency of sublingual absorption in orally exposed humans.

Arguments made by Gayrard et al. that “nanograms-per-milliliter” serum concentrations of BPA resulting from sublingual absorption are plausible in humans ignore key pharmacokinetic and exposure data and conflate issues of serum BPA concentrations with serum BPA/BPAG (BPA glucuronide) ratios.

For a given dose, bolus dosing yields peak concentrations of a parent compound that are higher than those with nonbolus dosing. Peak concentrations in mixed systemic serum following sublingual exposure cannot exceed those following intraveneous (iv) dosing. Therefore, in humans, peak systemic serum concentrations following sublingual exposure cannot be higher than those resulting from bolus iv administration of a given BPA dose. Scaling by dose from pharmacokinetic studies of nonhuman primates (Patterson et al. 2013), the human serum concentration of aglycone BPA immediately after an iv bolus administration of a dose equivalent to a 95th percentile of aggregate daily U.S. human exposure (0.22 μg/kg body weight; Lakind and Naiman 2010) would be < 0.1 ng/mL, which is below current quantification limits (LOQ) using state-of-the-art technology. Therefore, peak systemic serum concentrations of aglycone BPA following sublingual exposure/absorption of this dose would also be < LOQ. This result is consistent with the data of Gayrard et al. (2013) in which dog serum BPA concentrations would be ~ 0.07 ng/mL (i.e., < LOQ) when sampled from a site reflecting systemic exposure (e.g., leg vein). Therefore, in 95% of the U.S. population, serum aglycone BPA concentrations of nanograms per milliliter are not possible, even with complete sublingual absorption.

Several studies in the literature have reported BPA/BPAG ratios in human serum samples higher than those (< 1%) consistently observed in human and animal oral pharmacokinetic studies (Vandenberg et al. 2007). Gayrard et al. (2013) introduced a new hypothesis (i.e., sublingual exposure/absorption) to justify this discrepancy. Previously proffered hypotheses for high BPA and BPA/BPAG ratios in human serum samples included exposures 10,000 times higher (Vandenberg et al. 2007) than those measured in humans (Lakind and Naiman 2010), extensive dermal exposure/absorption (Vandenberg et al. 2013), and sample contamination (Teeguarden et al. 2011). Only the latter hypothesis is supported by ample evidence that contamination of blood samples is not only common, but difficult to avoid (Ye et al. 2013).

Finally, extrapolation of BPA levels from BPAG levels using the measured ratio of < 1% following ingestion of BPA is appropriate under conditions where BPA reaches the systemic blood via gastrointestinal tract absorption [e.g., food (see Teeguarden et al. 2011)]. Similarly, values of approximately 10–20% would be valid for serum levels 0–4 hr after iv dosing or complete sublingual absorption, when sampled from a site representing systemic exposure.

## References

[r1] Gayrard V Lacroix MZ Collet SH Viguié C Bousquet-Melou A Toutain PL 2013 High bioavailability of bisphenol A from sublingual exposure. Environ Health Perspect 121 951 956; 10.1289/ehp.1206339 23761051 PMC3734497

[r2] Lakind JS, Naiman DQ (2010). Daily intake of bisphenol A and potential sources of exposure: 2005-2006 National Health and Nutrition Examination Survey.. J Expo Sci Environ Epidemiol.

[r3] Patterson TA, Twaddle NC, Roegge CS, Callicott RJ, Fisher JW, Doerge DR (2013). Concurrent determination of bisphenol A pharmacokinetics in maternal and fetal rhesus monkeys.. Toxicol Appl Pharmacol.

[r4] Teeguarden JG, Calafat AM, Ye X, Doerge DR, Churchwell MI, Gunawan R (2011). Twenty-four hour human urine and serum profiles of bisphenol A during high-dietary exposure.. Toxicol Sci.

[r5] Vandenberg LN, Hauser R, Marcus M, Olea N, Welshons WV (2007). Human exposure to bisphenol A (BPA).. Reprod Toxicol.

[r6] Vandenberg LN, Hunt PA, Myers JP, vom Saal FS (2013). Human exposures to bisphenol A: mismatches between data and assumptions.. Rev Environ Health.

[r7] Ye X, Zhou X, Hennings R, Kramer J, Calafat AM. 2013 Potential external contamination with bisphenol A and other ubiquitous organic environmental chemicals during biomonitoring analysis: an elusive laboratory challenge. Environ Health Perspect 121 283 286; 10.1289/ehp.1206093 23458838 PMC3621191

